# Recommendations for Transitioning Young People with Primary Immunodeficiency Disorders and Autoinflammatory Diseases to Adult Care

**DOI:** 10.1007/s10875-024-01838-y

**Published:** 2024-12-17

**Authors:** Muskan Israni, Eliska Alderson, Nizar Mahlaoui, Laura Obici, Linda Rossi-Semerano, Helen Lachmann, Mojca Zajc Avramovič, Aurelien Guffroy, Virgil Dalm, Rachel Rimmer, Leire Solis, Carlota Villar, Andrew R. Gennery, Stephanie Skeffington, Julia Nordin, Klaus Warnatz, Anne-Sophie Korganow, Jordi Antón, Marco Cattalini, Stefan Berg, Pere Soler-Palacin, Mari Campbell, Siobhan O. Burns, the ERN RITA Transition Working Group Consortium, Luis Ignacio Gonzalez-Granado, Isabelle Meyts, Efimia Alataki-Papadopoulou, Reem Elfeky, Jadranka Kelečić, Jutte van der Werff ten Bosch, Zahir Amoura, Vanda Friman, Carsten Heilmann, Niall Conlan, Kristiina Aalto, Judith Sanchez-Manubens, Vito Sabato, Mikko Seppänen, Xavier Solanich, Antonella Insalaco, Rainald Zeuner, Alberto Tommasini, Elissaveta Naumova, Claudia Blattmann, Mary Slatter, Jiří Litzman, Pierre Philippet, Fabio Candotti, Olov Ekwall, Pavlina Kralickova, Markus Seidel, Clementina Canessa, Rik Schrijvers, Natasha Prescott, Ursula Holzer, Carmen Carreras, Maria Kanariou, Miloš Jeseňák, Constantinos Pitsios, Lisa Devlin, Peter Arkwright, Nico Wulffraat, Charalampia Papadopoulou, Bénédicte Neven, Lucia Baselli, Rosa Maria Dellepiane, Sinisa Savic, Nicholas Brodszki, Laia Alsina, Felipe Suarez, Viviana Moschese, Peter Jandus, Trine Hyrup Mogensen, Bjorn Runar Ludviksson, Elizabeth McDermott, Ruth Fritsch-Stork, Federica Barzaghi, Brindusa Capilna, Marina Folkers, Mary Keogan, Tim Niehues, Benson Ogunjimi, Maria Koliou, Guillaume Lefèvre, Stephen Jolles, Ana Méndez-Echevarría, Ewa Bernatowska, Rosie Hague, Jean-Christophe Goffard, Olivier Gilliaux, Saul Faust, Patricia Luck, Carine Wouters, Suzanne Elcombe, Dirk Holzinger, Maria Carrabba, Manuel Santamaria, Leif Hanitsch, Paul Brogan, Troels Herlin, Horst von Bernuth, Giovanna Fabio, Véronique Hentgen, Hanne Marquart, Susana Lopes da Silva, Terese Katzenstein, Tania Nicole Masmas, Olaf Neth, Filomeen Haerynck, Joke Dehoorne, Tania Amin, Georgia Hayward

**Affiliations:** 1 Department of Immunology, Royal Free London NHS Foundation Trust, London, UK; 2 Pediatric Immuno-Haematology and Rheumatology Unit, Necker Enfants Malades University Hospital, Assistance Publique-Hôpitaux de Paris (AP-HP), Paris, France; 3 French National Reference Center for Primary Immune Deficiencies (CEREDIH), Necker Enfants Malades University Hospital, Assistance Publique-Hôpitaux de Paris (AP-HP), Paris, France; 4 Fondazione IRCCS Policlinico San Matteo, Centro Per Lo Studio E La Cura Delle Amiloidosi Sistemiche, Pavia, Italy; 5 Department of Pediatric Rheumatology, National Reference Centre for Auto-Inflammatory Diseases and Amyloidosis of Inflammatory Origin (CEREMAIA), Bicêtre Hospital, Assistance Publique-Hôpitaux de Paris (AP-HP), Le Kremlin Bicêtre, France; 6 Division of Medicine, National Amyloidosis Centre, University College London, London, UK; 7 Department for Allergology, Rheumatology and Clinical Immunology, University Children’s Hospital Ljubljana, Ljubljana, Slovenia; 8 Department of Clinical Immunology and Internal Medicine, National Reference Center for Systemic Autoimmune Diseases (CNR RESO), Tertiary Center for Primary Immunodeficiency, Hôpitaux Universitaires de Strasbourg, 67000 Strasbourg, France; 9 Université de Strasbourg, INSERM UMR - S1109, 67000 Strasbourg, France; 10 Department of Internal Medicine, Division of Clinical Immunology, Erasmus University Medical Center, Rotterdam, Netherlands; 11 Department of Immunology, Erasmus University Medical Center, Rotterdam, Netherlands; 12 Rare Autoinflammatory Conditions Community – UK (RACC – UK), Eynsham, UK; 13 International Patient Organisation for Primary Immunodeficiencies (IPOPI), Brussels, Belgium; 14 Barcelona PID Foundation, Barcelona, Catalonia Spain; 15 Paediatric Haematopoietic Stem Cell Transplant Unit, Great North Children’s Hospital (GNCH), Royal Victoria Infirmary, Queen Victoria Road, Newcastle Upon Tyne, NE1 4LP UK; 16 Translational and Clinical Research Institute, Faculty of Medical Sciences, Newcastle University, Newcastle Upon Tyne, NE2 4HH UK; 17 Irish Vasculitis Organisation, Tipperary, Ireland; 18 Center for Chronic Immunodeficiency, Medical Center - University of Freiburg, Faculty of Medicine, University of Freiburg, Freiburg, Germany; 19 Department of Rheumatology and Clinical Immunology, Division of Immunodeficiency, Faculty of Medicine, Medical Center - University of Freiburg, Freiburg, Germany; 20 Department of Pediatric Rheumatology, Sant Joan de Déu Hospital, Pediatric Immune Dysfunction Disease Study Group (GEMDIP), Institut de Recerca Sant Joan de Déu, Barcelona, Spain; 21 Pediatrics Clinic, University of Brescia and ASST Spedali Civili Di Brescia, Brescia, Italy; 22 Department of Pediatrics, Institute of Clinical Sciences, The Sahlgrenska Academy, University of Gothenburg, Gothenburg, Sweden; 23 Department of Pediatrics, Queen Silvia Children’s Hospital, Sahlgrenska University Hospital, Gothenburg, Sweden; 24 Pediatric Infectious Diseases and Immunodeficiencies Unit, Children’s Hospital, Vall d’Hebron, Barcelona Hospital Campus, Barcelona, Catalonia Spain; 25 Jeffrey Modell Diagnostic and Research Center for Primary Immunodeficiencies, Barcelona, Catalonia Spain; 26 University College London Institute of Immunity and Transplantation, London, UK

**Keywords:** Transition, recommendations, inborn errors of immunity, primary immunodeficiency disorders, autoinflammatory diseases

## Abstract

**Purpose:**

Significant improvements in the prognosis for young patients with Primary Immunodeficiency Diseases (PID) and Autoinflammatory Disorders (AID), which together make up the majority of Inborn Errors of Immunity (IEI), have resulted in the need for optimisation of transition and transfer of care to adult services. Effective transition is crucial to improve health outcomes and treatment compliance among patients. Evaluations of existing transition programmes in European health centres identified the absence of disease-specific transition guidelines for PID and AID, as a challenge to the transition process. This research aimed to establish expert consensus statements for the transition of young patients with PID and AID to adult services.

**Methods:**

This project used the Delphi method to establish mutual agreement for the proposed recommendations. A draft set of statements was developed following a literature review of existing transition programmes. Then the ERN RITA Transition Working Group convened to review the drafted recommendations and develop them into a survey. This survey was circulated among healthcare professionals to determine consensus using a five-point Likert scale, with the level of agreement set to 80% or greater. Statements that did not reach consensus were revised by the Working Group and recirculated among respondents.

**Results:**

The initial survey received 93 responses from 68 centres across 23 countries, while the following survey outlining revised recommendations received 66 responses. The respondents agreed upon recommendations detailing the structure and administration of transition programmes, collaborative working with social systems, and contraindications to transfer of care.

**Conclusion:**

This paper sets out a comprehensive set of recommendations to optimise transitional care for PID and AID.

**Supplementary Information:**

The online version contains supplementary material available at 10.1007/s10875-024-01838-y.

## Introduction

Inborn Errors of Immunity (IEI) refer to a group of rare disorders characterized by elevated, poor or absent function in components of the innate and/or adaptive immune system. Primary immunodeficiencies (PID) and Autoinflammatory Diseases (AID) represent the majority of known IEI. Substantial advancements in diagnostic tools and treatment of IEI have resulted in reduced delay to diagnosis and improved life expectancy of young patients in recent years [1, 2]. Consequently, there has been a growing emphasis on ensuring optimal transition of care and long-term follow-up of young patients with IEI in adult care services [3, 4].

Transition—as opposed to transfer of care—refers to the “purposeful, planned process that addresses the medical, psychosocial, and educational/vocational needs of adolescents and young adults with chronic physical and medical conditions as they move from child-centred to adult-oriented health care systems” [5]. The need for a holistic, standardised transition programme within services is further demonstrated by research suggesting poorer health-related quality of life in patients with IEI compared to the general population [6, 7]—with notable gaps identified not only in patients’ physical functioning, but also their social, emotional, and school functioning [6, 8].

Dedicated transition programmes within health services have been observed to improve young patients’ disease and self-management knowledge, health-related quality of life, and health outcomes, among a wide range of chronic conditions such as diabetes mellitus [9], cystic fibrosis [10], and juvenile idiopathic arthritis [11]. However, among Allergist/Immunologists treating PID specifically, only a quarter of practitioners surveyed reported satisfaction with their existing transition practices [12]. Findings from this survey suggest that existing generalised transitional care resources are not sufficient to address the needs of patients with IEI due to the heterogeneity of their condition – with close to half of all practitioners expressing a demand for condition-specific guidance. The relative lack of adult specialists treating IEI also poses a challenge to effective transition and transfer of care from paediatric services [13]. Individual centres have drawn upon their experience of transitioning young patients with PID [14, 15] and AID [16] to adult care to share practices effective in optimising health outcomes and follow-up in adult services – such as the appointment of dedicated transition personnel, access to psychosocial support, and establishment of formal transition programs.

The European Reference Network on Rare Immunodeficiency, Autoinflammatory and Autoimmune Disease (ERN-RITA) Transition Working Group was set up in 2017 to develop standardised, best-practice guidelines for the transition of young people with IEI, encompassing both PID and AID, from paediatric to adult care. This Working Group comprises multidisciplinary healthcare professionals from paediatric and adult teams with expertise in managing PID and AID, as well as Patient Experts.

In the first phase of this study (June 2018—December 2019), a survey was circulated to paediatric centres managing PID and/or AID across Europe to examine current practices for transition to adult healthcare services and identify gaps and barriers to effective transition [3]. A key finding of the survey was the lack of national disease-specific guidelines for transition for these conditions. As international guidelines are also lacking, the ERN RITA Transition Working Group sought to develop consensus-based guidelines to standardise and optimise transition care for these specific rare disease groups.

## Methods

This research used the Delphi method due to its efficiency in harnessing the opinions of expert panellists across a large geographical patch and establishing consensus on proposed policies in healthcare settings [17]. This method was also chosen in line with guideline development requirements for ERN-RITA and the European Society for Immunodeficiencies (ESID) Clinical Working Party.

A systematic literature review was performed from June to September 2021 to explore existing transition programmes and recommendations for young people with PID and AID. The following keywords were searched on PubMed and Google Scholar databases: ‘transition from paediatric to adult care’ or ‘transition of care’ AND ‘young adults or adolescents’ AND ‘primary immunodeficiency disorders’ or ‘inborn errors of immunity’ AND ‘autoinflammatory diseases’. All papers were scored for their level of evidence (Supplementary Table 1) which was used to inform grade of recommendation. The findings from the literature review and the ERN RITA survey were used to develop a draft set of statements informing the process and administration of transitional care. For Round One of the Delphi process (September 2021), the ERN RITA Transition Working Group met virtually to review and revise the draft set of recommendations. The draft of proposed recommendations was also emailed to Working Group members following the meeting to offer feedback. The Transition Working Group then reconvened to agree upon a final list of proposed transition protocols for patients with PID or AID and develop a survey detailing the same.

In Round Two of the Delphi process, the survey was circulated among healthcare professionals through IPOPI (N = 231) and INGID (N = 249) registries and professional networks in psychology (N = 5). Specifically, clinicians from all centres in Europe listed in the IPOPI PID Life Index website [52] with a listed email address were contacted. This includes a mix of academic medical centres and other hospitals who provide services for children and adults with PID. Many of these also care for children and adults with AID. The email listed was typically for a senior physician in the service. Other centres known to care for paediatric or adult PID or AID patients through professional networks of the ERN RITA transition Working Group members were also contacted. We did not specifically distribute the survey broadly to other medical specialties, for example Haematology or Rheumatology services, who may see some patients with PID or AID within their patient cohorts. Primary care providers were not included in the survey.

Participants were asked to rate each statement on a 5-point Likert scale (Strongly Agree, Tend to Agree, Neither Agree nor Disagree, Tend to Disagree, Strongly Disagree). The level of agreement was set at 80% or greater to reach consensus and adoption in the guidelines.

In Round Three of the Delphi Process, experts in the Transition Working Group were requested in a virtual meeting and via email to revise and/or omit recommendations that did not achieve consensus in Round Two. A revised set of statements was then re-circulated among responding health centres in the initial survey to establish consensus.

## Results

**Fig. 1:**
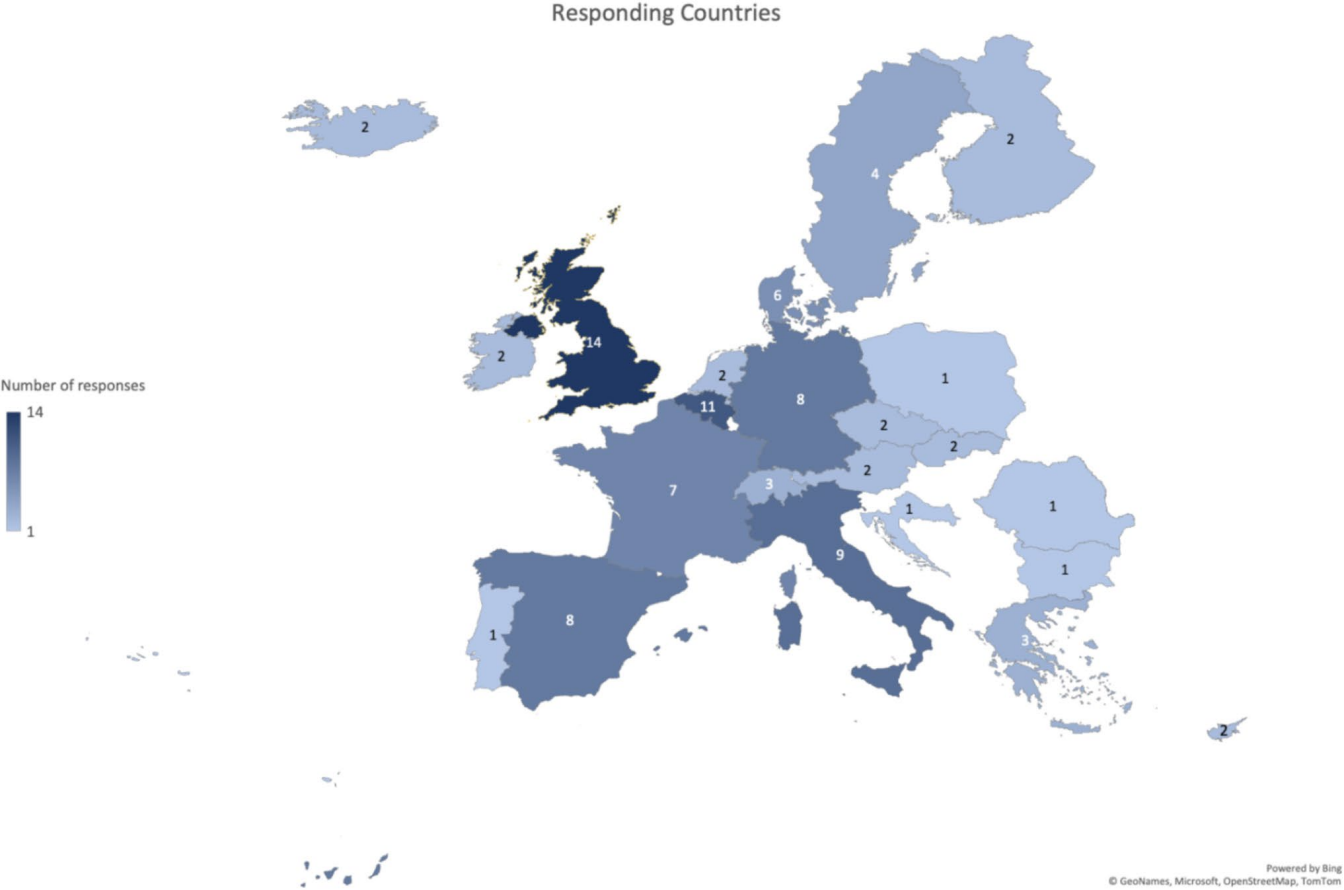
European map of the participants in the surveys

In the initial survey detailing proposed recommendations for transitional care, 229 individuals were contacted, and 93 responses (41%) were received from 68 centres across 22 countries (Fig. 1). Of the 93 responding centres, 58 responses were received from services caring for both PID and AID, 23 responses from PID centres alone, and 12 responses from AID centres. 90/93 responders were physicians. 24/93 responders reported caring only for adult patients only, 45/93 for paediatric patients only and 24/93 cared for both. 60 of the 93 responders (65%) surveyed reported transferring less than 10 young patients to adult services each year (Supplementary Fig. 1). No significant differences were seen in the patterns of response when analysed according to whether responders cared for AID, PID or both or whether responders cared for paediatric or adult patients or both Supplementary Fig. 2a, b). There were also no significant differences in responses when analysed by centre size assessed by whether < 5, 5–10 or > 10 patients were transferred to adult care per year (Supplementary Fig. 2c). Finally, we also did not find differences in responses from countries which were poorly represented (only 1–2 responders) compared with countries that were well represented (3 or more responders) (Supplementary Fig. 2d).

In the next round, the survey detailing the revised set of recommendations was sent only to initial responders and received a total of 66 responses (70% of initial responders). The survey responses represent clinicians (medical, nursing, and psychology professionals) from paediatric and adult services—with 46% of respondents working in paediatric PID and AID services, 27% working in adult services, and a further 27% working in services following up paediatric and adult patients with PID and AID.

The proposed set of statements guiding transitional care that achieved consensus are categorised into the following subsections:

### Transition Process

Following two rounds of surveys, 15 of the proposed statements concerning the facilitation of safe and smooth handover of care to adult services achieved consensus with the level of agreement ranging from 84 to 100% (Table 1). These recommendations propose gradually building young patients’ preparedness through measures such as starting the transition process early and offering communication with both paediatric and adult care teams in the year before transfer. The recommendations also include tailoring transition programs to the young patients’ needs—reviewing diagnostic evaluations and management plans prior to transfer and ensuring multidisciplinary follow-up as appropriate. Five statements did not reach consensus (level of agreement ranging from 57% of respondents to 78%) (Supplementary Table 2).

**Table 1:** Recommendations for the process of transition

Recommendations for the process of transition	Level of evidence	Level of agreement	Grade of recommendation
The transferring centre should organise ‘formal transition partners’ with integrated care pathways for smooth transition to adult care services	4	96%	D
Discussion about and planning of transition should ideally start at the paediatric centre before patients are 16 years old	4	90%	D
The process of transition should be adapted for individual clinical and developmental needs	5	100%	D
The transition process should not have a time-limit; the duration must be suited to the patient’s requirements	5	87%	D
Where possible, young patients should have a period of joint follow-up by the paediatric and adult clinics in the year prior to transfer	5	84%	D
Transition care should include consideration of all extra-immunological manifestations of the disease	5	99%	D
Young patients should have access to multidisciplinary care suited to address their multi-morbidities during transition and after transfer	5	100%	D
It is the responsibility of the lead paediatric clinician to clarify who is in charge of monitoring specific issues during the transition period	5	90%	D
A diagnostic re-evaluation should be considered for young patients without a definitive diagnosis prior to leaving the paediatric service	5	85%	D
Young patients should not leave the paediatric service without a re-evaluation of the management plan	5	93%	D
Young patients and their families should be provided with verbal and written information about the transition process and what it involves	5	86%	D
Where possible, young patients should have the opportunity for a (virtual) walk-through of the adult service to build familiarity with the site and its staff prior to transfer	4	85%	D
Ideally, patients should have appointment schedules that minimise interruptions to their school/daily routine (e.g. during holiday periods)	5	87%	D
Patients should have continued access to a paediatric team until they are seen in adult services	5	99%	D
Young adults and their caregivers should have access to the referring paediatric physician, as well as the adult physician, via telephone or email for any questions during the transition period when paediatric and adult care overlap	5	87%	D

### Administration of Transition

Following two rounds of surveys, 14 statements regarding the effective and efficient implementation of transition programs reached consensus with the level of agreement ranging from 84 to 100% (Table 2). These guidelines recommend prompt handover of all relevant patient information including medical records, involvement in past or current research, and follow-up plans prior to the young person’s first appointment in adult services. Respondents also recommended that transition programs take an interdisciplinary approach—linking in young patients and caregivers with relevant healthcare professionals and community resources such as patient advocacy groups and youth workers to support with illness and adolescence-related concerns. Four statements did not reach consensus (level of agreement ranging from 70% of respondents to 77%) (Supplementary Table 2).

**Table 2:** Recommendations for the administration of transition programmes

Recommendations for the administration of transition programmes	Level of evidence	Level of agreement	Grade of recommendation
Centres transferring and receiving patients should have a defined transition policy or program in place	5	99%	D
Centres transferring or receiving patients should have a dedicated staff member/entity responsible for coordinating transition and transfer of care across services	4	89%	D
Paediatric and adult medical staff involved in the transition process should be trained in issues relating to adolescence and transition	4	98%	D
Young patients with IEI should ideally be discussed in multidisciplinary meetings to ensure personalised transition programmes and holistic care	5	94%	D
Members of the paediatric and adult multidisciplinary teams involved in transition should undergo training in understanding IEI, their clinical presentations, and patients’ comorbidities arising from IEI or associated treatments, to ensure best management	5	99%	D
A paediatric and/or adult specialist nurse may support smooth transition by keeping up-to-date on their health status after transfer and answering questions from the patient and their family	5	96%	D
There must be effective communication between paediatric and adult care teams—with transfer of all medical records taking place in advance of the first appointment at the adult service	4	99%	D
Relevant information regarding research involvement, research results and ongoing clinical trials should be transferred with the medical records	5	97%	D
Young patients enrolled in active clinical trials should have appropriate provisions made for continuity of trial monitoring after transfer to an adult centre	5	98%	D
Patient records sent to the adult service must clearly state involvement in ongoing clinical trials and all follow-up plans and ensure continuity of trial involvement	5	100%	D
The results of relevant clinical trials assessments should be sent back to the original care team	5	89%	D
Where possible, patients and families should be signposted to online informational resources, while warning them to only consult reliable websites	5	84%	D
The transition process should offer young patients the opportunity to interact with other patients of the same age (or peers with the same disease) by:			
a. Signposting them to community resources like patient advocacy groups	5	87%	D
b. Organising or signposting to age-appropriate educational events for young patients and their parents	5	84%	D
Young patients should be supported in accessing community resources by a team of multidisciplinary healthcare professionals such as social workers and youth workers	5	92%	D

### Transition Appointments

All eight proposed statements concerning the organisation of transition consultations to gradually increase the young patients’ involvement in their healthcare reached consensus with the level of agreement ranging from 90 to 100% (Table 3). These recommendations propose holding joint transition appointments with paediatric and adult clinicians to build the patients’ familiarity with the adult care team. It is also recommended to support greater patient autonomy through 1:1 consultations with physicians, seeking their consent to share information with caregivers as appropriate, and asking them to re-consent to research they have been enrolled in by caregivers.

**Table 3:** Recommendations for the structure of transition appointments

Recommendations for the structure of transition appointments	Level of evidence	Level of agreement	Grade of recommendation
Joint transition consultations should be held with patients, their families, and healthcare professionals from the paediatric and adult teams present for smooth handover of care	5	92%	D
In line with local legal frameworks, the young patient should be offered dedicated parent-free consultations or dedicated 1:1 time for physicians and patients during consultations in the years prior to transfer to the adult centre. This should include chaperoned physical examinations	5	90%	D
Parents should be supported to decrease their involvement in care through discussions about the need to support the patient’s independence and autonomy in disease management	4	95%	D
Time should be dedicated to answering questions from family members during each consultation	5	94%	D
The young patient should be made aware about the need for consent to share information with their family/caregivers, following transfer, unless the young patient is unable to provide informed consent – such as in the event of learning disabilities	5	97%	D
Young patients should be reinformed about studies/biobanks that they have been enrolled in by parents/carers and affirm assent, and where necessary re-consent should be taken. This should be formally documented with details of the research study	5	99%	D
It should be clarified to the young patient and their caregivers that care and prescribing treatment remain with the paediatric centre until the patient has been seen at the adult centre	5	100%	D
It should be confirmed with the young patient that active medications can be continued in the adult centre	5	97%	D

### Content of Transition Programmes

Sixteen statements regarding the development of transition programmes reached consensus with the level of agreement ranging from 85 to 100% (Table 4). These guidelines suggest using standardised measures of transition readiness to inform personalised and age-appropriate care plans offering patients medical, psychosocial, educational, and employment guidance to manage the impact of their illness on quality of life. It is also recommended that transition programmes include education on the nature of the illness and treatment options, self-administration of medication, and management of disease flare-ups to promote greater independence among young patients. One statement and three statements did not reach consensus (level of agreement ranging from 65% of respondents to 75%) (Supplementary Table 2).

**Table 4:** Recommendations for the content of transition programmes

Recommendations for the content of transition programmes	Level of evidence	Level of agreement	Grade of recommendation
Young adult patients, their family, and multidisciplinary clinicians should collaboratively develop a personalised transition plan that is:			
a. Designed in accordance with specific training needs and knowledge gaps of the patient b. Accommodating of the maturational, social, and personal changes accompanying adolescence c. Culturally sensitive	5 5 4	86% 90% 87%	D D D
There should be an emphasis on person-centred holistic care tailored to the patient’s needs and age. For instance, access to psychosocial support, and educational or vocational guidance should be offered as and when appropriate	5	95%	D
The healthcare teams (at paediatric and adult centres) must routinely monitor patients to screen for complications during transition (e.g., organ damage, uncorrected manifestations, incomplete immune reconstitution, chronic graft vs host disease, etc.)	5	92%	D
Using written and verbal information, the transition process should include education of the young adult on:			
a. Nature of their disease b. The genetic basis of their disease or possible investigations, if appropriate c. The heritability of their condition, family-planning guidance and genetic counselling where appropriate d. Treatment options and their long/short-term effects e. Gene and cell therapy f. How to manage a disease flare-up g. Comorbidities h. Disease progression i. How their condition and associated treatments impact sexual and reproductive health and function including fertility, pregnancy, and contraception j. Quality of life with IEI k. Vocational planning l. Support resources (e.g. patient support groups) m. Maintaining a healthy lifestyle	4 5 5 5 5 5 5 5 5 5 5 5 5	95% 95% 95% 95% 92% 95% 96% 95% 94% 92% 86% 92% 97%	D D D D D D D D D D D D D
Young adults should be supported for increased self-management of their condition through education and training in:			
a. Symptom/complication monitoring b. Administering medication c. Booking/managing consultations d. Contacting physicians in the event of sickness	5 5 5 4	97% 97% 96% 96%	D D D D
Increased self-management can also be encouraged by addressing young patients themselves—rather than their parents/caregivers—during consultations	5	100%	D
During the transition process, routine assessments should be performed using standardised measures (where available) of:			
a. Quality of life b. Functional status c. Psychological functioning	5 5 5	85% 88% 86%	D D D
Adherence to treatment must be evaluated during the transition stage and young adult patients must receive guidance on the importance of medication adherence and consequences of non-compliance	5	98%	D
Psychological support must be accessible to patients at the time of transition and in the adult service to manage emotional concerns arising from illness and/or transition to adult service	5	98%	D
Families and medical teams should progressively support young adults’ involvement in Shared Decision Making regarding clinical care through guided decision-making during the transition process	5	94%	D
The transitioning team should ensure that young adult patients have access to age-appropriate care from a primary care physician	5	95%	D
The young person should be offered educational and vocational guidance, and work with the transition team on a personalised professional plan adjusted as per the requirements/restrictions imposed by their condition	4	85%	D
Patients must have continued access to safe, local, and convenient long-term therapeutic intervention, with appropriate supervision and monitoring	5	97%	D
The transferring paediatric team should outline a care plan for the patient that includes an active and inactive problem list and plans for patient-specific routine follow-up monitoring	5	95%	D
The transition plan should include vaccination history and plans of a vaccination course in adulthood where special consideration is required	5	98%	D
Where appropriate due to limited life expectancy, the transition team should support young patients and their families through decision-making regarding end-of-life planning	4	86%	D

### Managing Other Systems

Three statements proposing integrated health and social care for young patients reached consensus with the level of agreement ranging from 90 to 95% (Table 5). These recommendations suggest informing young people of their patient rights and supporting them to access reasonable accommodations in educational and workplace settings. One statement did not reach consensus (level of agreement 55%) (Supplementary Table 2).

**Table 5:** Recommendations for managing other systems in the transition process

Recommendations for managing other systems in the transition process	Level of evidence	Level of agreement	Grade of recommendation
Ideally, the paediatrics team should identify local, national/international networks to support/share care of patients with rare disorders	5	95%	D
The young patient should be made aware of their country-specific rights in the healthcare setting	5	90%	D
The young patient should be supported to inform their school or educational facility and/or workplace about their condition and treatments—for example to ensure appropriate adjustments and facilitate access to medical appointments	5	91%	D

### Contraindications to Transfer of Care

All four statements outlining circumstances in which transfer of care to adult services may be delayed or avoided reached consensus with the level of agreement ranging from 83 to 90% (Table 6). It is recommended that young patients not be transferred during critical illness, infection episodes or with unstable disease, particularly if the adult service is not equipped to manage the patients’ disease presentation, progression, and multimorbidities. Thus, transfer via the emergency department should be avoided. It is also recommended that young patients should be monitored for a period of disease stability leading up to transfer of care. There may be exceptions to this, for example where specific therapies need to be initiated (such as haematopoietic stem transplant) that would be more appropriately delivered at the adult service.

**Table 6:** Contraindications to transfer of care to adult services

Contraindications to transfer of care to adult services	Level of evidence	Level of agreement	Grade of recommendation
Transition should be delayed for specific circumstances such as critical illness due to severe invasive and/or systemic infections, autoimmune or inflammatory manifestations	5	90%	D
Young patients presenting with unstable disease should be stabilised prior to transfer	5	87%	D
Where possible, for patients with severe IEI, transition should be delayed or avoided if the adult service is not equipped to handle potential severe progression of disease and frequent clinical instability of patients	5	90%	D
A delay in transition may be necessary for young patients enrolled in active clinical trials where continuity of trial monitoring after transfer to an adult centre is not in place	5	83%	D

## Discussion

Improved prognoses for young patients with IEI have brought about the challenge of safe, effective, and efficient transfer of care to adult health services [18–22]. This paper outlines recommendations for best practice guidance for the varied elements of the transition process—from structuring transition clinic appointments to the content of educational programmes for young patients with PID or AID, which make up the majority of IEI.

In doing so, this paper sets out a transition pathway following three key principles:

### Bolstering Young Patients’ Relationship with the Adult Care Team

Prior research has observed the close relationships between young patients with IEI and their caregivers and paediatric centres to be a barrier impacting transition to adult services [23, 24]. The prospect of leaving the care of trusted paediatric care professionals, as well as the lack of familiarity with adult care teams both hinder successful transfer of care [23–27]. Therefore, the proposed guidance recommends that paediatric centres support patients in extending their confidence to adult care teams by establishing formal transition partners and offering joint appointments during the transition period.

#### Supporting Young Patients’ Self-Efficacy

Young patients with chronic illnesses such as IEI have frequently reported feeling ‘unprepared’ for the transition to adult care services—describing deficits in their understanding of their illness and their medication, dependence upon parents to manage their healthcare needs, and anxieties about assuming increased responsibility for their health management [23, 24, 26–28]. Hence, the proposed transition guidelines recommend measures such as implementing education programmes to boost patients’ health literacy, and skills in self-administering medication and managing disease flare-ups. The proposed guidance also recommends progressively augmenting young patients’ autonomy by addressing them directly in appointments, offering 1:1 consultations with physicians without caregivers present, and helping caregivers support the young adult patient in increasing self-management. Thus, the proposed measures aim to ‘upskill’ young patients by addressing their knowledge gaps and empower them to participate in Shared Decision Making with clinicians during the transition period [29]. As outlined in the recommendations, the delivery of patient education may be enabled through access to transition-focused specialist nurses prior to transfer of care, offering person-centred care wherein clinical teams tailor information shared during appointments to meet the knowledge gaps of young patients, and through effective signposting to community resources and patient information leaflets [30]. The International Patient Organisation for Primary Immunodeficiencies has developed informational videos and leaflets for physicians and patients about IEI and their management in multiple languages (https://ipopi.org/publications/leaflets/). The Immune Deficiency Foundation has also developed a Patient and Family Handbook for Primary Immunodeficiency Diseases (https://primaryimmune.org/search?s=handbook) available in English and Spanish with information about the diagnosis, treatment, and management of PID, including chapters for specific disorders. National patient organisations in the European Union and globally also produce valuable information in their national language for patients and professionals. Our recommendations regarding patient education are supported by existing literature reporting the benefits of patient education groups in augmenting independence and health-related quality of life among young people with IEI and other chronic conditions [31–33].

#### Offering Holistic and Integrated Care to Young Patients

A noted challenge to the effective transition of young patients with IEI and multimorbid conditions is the ‘fragmentation’ of healthcare teams [3, 23]. Young patients have reported experiencing their care to be ‘scattered’—needing to access separate services to manage distinct comorbid concerns [23]. Hence, the proposed guidance recommends that transferring centres identify appropriate, multidisciplinary adult services that can address patients’ individual clinical needs. It is also recommended that young patients should have continued contact with paediatric services, e.g., by phone or email, for any questions or concerns that may arise until transfer of care to adult services.

The guidelines proposed in this paper also recommend that young patients are offered holistic care—with access to timely psychosocial support, peer support, educational and vocational guidance, and patient advocacy groups. This is of particular significance due to the observed reciprocal relationship between chronic physical health conditions and mental health among young patients [24, 34]. Patients often describe poorer psychological wellbeing and social functioning than the general population—reporting feelings of ‘not fitting in’ or ‘wanting to give up’ [6, 8, 34]. These factors have been further observed to adversely impact physical health outcomes—with patients with comorbid IEI and mental health conditions reporting poorer treatment compliance and appointment attendance due to decreased motivation [24, 34]. Research exploring the perspectives of young patients with IEI demonstrates some demand for peer spaces to support psychological wellbeing and social inclusion [18, 35–37].

Initial clinical trials evaluating transition programmes for chronic illnesses such as diabetes, cystic fibrosis, and inflammatory bowel disease show promising effects on appointment attendance, transition readiness, and health-related distress among young patients [9, 38, 39]. However, there is limited data on outcomes of transition programmes for young patients with IEI – with only one paper reporting that it was not associated with an increase in unplanned healthcare utilization [15]. The studies that were used to develop the guidelines were expert opinion papers or small case series (Level of Evidence 4 or 5) [40] However, these guidelines are in line with the consensus statements for PID that were published recently by the Italian Primary Immunodeficiency Network [41] and the more general guidelines for transition for all mental and physical health services [42]. Additionally, the proposed guidance recommends offering comprehensive care that accounts for patients’ developmental and psychosocial needs to decrease rates of disengagement from follow-up care and prevent worsening of health outcomes [25, 27]. These efforts are further in alignment with overarching healthcare policies that aim to improve continuity of care for chronic health conditions, such as current NICE guidelines and the NHS Long Term Plan (UK) [42, 43] and the German Society for Transition Medicine Guidelines [44]. For instance, the proposed transition protocols also recognise the transition process as distinct from the act of transfer, and recommend offering age-appropriate, person-centred care to address adolescent patients’ individual clinical and developmental needs.

There were a number of notable topics that did not reach consensus (Supplementary Table 2). For example, the proposed statement to support young patients in navigating insurance coverage concerns did not achieve consensus, and thus were not adopted into the best-practice guidelines. This is in contrast to research involving participants in the USA which identify maintaining patients’ health insurance coverage as a major factor impacting transition of care [12]. It is important to note that the guidelines produced in this study represent the perspectives of European health centres which predominantly have access to universal healthcare, and so may be limited in its understanding of challenges to transition in countries without universal coverage. Additionally, there was lack of agreement about the use of digital tools to enable a smooth transition. In keeping with findings from prior research [12], the consensus statements largely promote the use of online resources to support young patients’ education, but there was lack of consensus around their optimal use for communication with patients (Supplementary Table 2). Specifically, there was disagreement around the use of smartphone or computer apps for patients to use as an ‘Electronic Health Record’ (for example of disease activity, complications, hospitalisations, treatments, and adherence) and around the use of text messages or emails, as opposed to postal mail, to communicate with young patients. Lack of agreement on the use of digital communication tools may be due to concerns around maintaining data security [45] and it is advisable for health services to seek explicit consent from young patients and consult their local legal frameworks if they incorporate email or text message technology into their transition programmes. While, the majority of respondents agreed that young patients’ treatment compliance should be monitored due to the observed decline in medication adherence during the transition period [14, 46, 47], there was no consensus among respondents about the use of tools including electronic apps to evaluate adherence. Despite this, there are existing systematic reviews demonstrating the effectiveness of electronic monitoring tools (such as devices that record the time and date of medication-taking) to improve adherence among patients with chronic conditions [48].

We acknowledge that there are a number of barriers to implementation of these guidelines. Differences in the organisation of paediatric and adult services between geographical regions will impact the availability and location of formal transition partners. For example, in some countries dedicated Immunology services do not exist for adult patients who are cared for by a range of other specialists including Rheumatology, Respiratory, Infectious Diseases and Haematology physicians. Surveys of transition practice for PID and AID have already highlighted difficulty in identifying specialist adult centres as an important problem both in Europe and Southeast Asia suggesting that this is a widespread issue [3, 49]. Even where both paediatric and adult services are available for transition, co-ordinating a transition process that is tailored to the needs of individual patients is a challenge. For example, many paediatric hospitals have an upper age-limit for in-patient and out-patient care of paediatric patients which make it difficult to delay transition even when patients are clinically unstable, or continuity of care cannot be guaranteed.

Other infrastructural barriers to implementation include insufficient resourcing of services to appoint appropriate specialist nurses, transition co-ordinators and administrative staff, availability of psychology support and processes to facilitate smooth transfer of health record information between centres. Different approaches are likely to be required in different regions to address organisation and resourcing issues to enable good transition but include promoting specialty training, influencing health care providers and working with patient groups to raise awareness of the need for robust paediatric and adult services for life-long care of PID and AID. In addition, the majority of responses in our Delphi were from Western Europe, with a large number from the United Kingdom and therefore future research is needed to fully understand the difficulties in implementing these recommendations in different health care settings and across geographical regions.

These guidelines were co-produced using the experiential knowledge and expertise of patient representative groups alongside paediatric and adult healthcare professionals. This is of significance as it is widely recognised that involving patients in the development and execution of healthcare services helps better address their needs and expectations, and resultantly improves health outcomes, patient satisfaction, and cost-efficiency in services [50]. Nonetheless, there is scope for further partnership with patients with lived experience in the development of transition protocols. Patient experts in the ERN RITA Transition Working group were consulted whilst drafting the proposed set of statements for transitional care from paediatric to adult services. However, the survey seeking to establish consensus on the outlined guidelines was primarily circulated among healthcare professionals through IPOPI, INGID, and psychology networks. Hence, it is recommended that future research examine patients’ perspectives, feedback, and acceptance of the proposed transition guidance.

It is also important to note that the best-practice guidance put forward in this paper primarily represents the perspectives of paediatric and adult medical professionals. The survey with proposed statements was circulated among a small number of psychologists working in immunology health services, and the response rate received from nursing networks was low. This is significant due to the wide cohort of healthcare professionals involved in transitioning young patients from paediatric to adult health services [3], and further research updating the outlined transition guidance should consult the perspectives of multidisciplinary health professionals.

Here we present the European consensus guidelines for the transition of young patients with PID and AID. A follow up study is required to audit the implementation of these guidelines and barriers to this. Future research should also evaluate the efficacy of these recommendations using standardised measures such as TRAQ [51] to monitor young patients’ preparedness prior to and following engagement in transition programmes.

## Supplementary Information

Below is the link to the electronic supplementary material.

Supplementary Table 1 (DOCX 15.4 KB)

Supplementary Table 2 (DOCX 16.2 KB)

Supplementary Fig. 1 (DOCX 16 KB)

Supplementary Fig. 2 Heatmaps for all statements where agreement was >80% and <90%, sorted by responder category (left and column). The following categories are shown:
(a) Disease category cared for: purple = autoinflammatory disease, dark blue = primary immunodeficiency, light blue = both
(b) Age of patient seen: purple = adults, dark blue = children, light blue = both
(c) Number of patients transferred to adult care per year: purple = <5, light grey = 5 to 10, light blue = >10; dark grey= unknown
(d) Country representation: purple = poorly represented with 1-2 responders; better represented with >/= 3 responders.
For (a)-(d):
Top panels: rows represent individual responders; columns represent individual questions. Responses are coloured as follows: strongly agree = dark green; tend to agree = light green; neither agree nor disagree= yellow; tend to disagree= orange; strongly disagree=red; white = question not answered.
Bottom panels: rows represent average response score for responders divided by specific categories represented in the top panels. Average response scored are coloured as follows: strongly agree = dark green; tend to agree = light green; neither agree nor disagree= yellow; tend to disagree= orange; strongly disagree=red.
Strongly agree = average score for the category >/= 1.5; tend to agree = average score 1.49 to 0.5; neither agree nor disagree = average score 0.49 to -0.49; tend to disagree = average score -0.5 to -1.49; strongly disagree= average score </= -1.5. Statistical analysis for category comparison used the t-test; p<0.05 considered statistically significant. (DOCX 188 KB)

## Data Availability

The data that support the findings of this study are not openly available due to reasons of sensitivity and are available from the corresponding author upon reasonable request. Data are located in controlled access data storage at University College London.
