# Supplementary material for: Recommendations for Transitioning Young People with Primary Immunodeficiency Disorders and Autoinflammatory Diseases to Adult Care
Source: J Clin Immunol. 2024 Dec 17;45(1):57. doi: 10.1007/s10875-024-01838-y (PMC11652586; doi:10.1007/s10875-024-01838-y)
Supplement: Supplementary file 1 — Supplementary Table 1 (DOCX 15.4 KB) [file 10875_2024_1838_MOESM1_ESM.docx]

Supplementary Table 1. Papers identified in the literature review.

| **Title** | **Digital Object Identifier (DOI)/Link** | **Level of Evidence** |
| --- | --- | --- |
| Adolescents and young adults (AYAs) affected by chronic immunological disease: A tool-box for success during the transition to adult care | 10.1016/j.clim.2018.10.010 | 5 |
| Transition from Pediatric to Adult Care by Young Adults with Chronic Granulomatous Disease: the Patient’s Viewpoint | 10.1016/j.jadohealth.2017.06.017 | 4 |
| Physical health conditions and quality of life in adults with primary immunodeficiency diagnosed during childhood: A French Reference Center for PIDs (CEREDIH) study | 10.1016/j.jaci.2016.08.027 | 4 |
| Primary Immunodeficiency Disease: A Model for Case Management of Chronic Diseases | 10.1097/NCM.0b013e3181b5dec4 | 5 |
| Advances in the Care of Primary Immunodeficiencies (PIDs): from Birth to Adulthood | 10.1007/s10875-017-0401-y | 5 |
| Consensus of the Italian Primary Immunodeficiency Network on transition management from pediatric to adult care in patients affected with childhood-onset inborn errors of immunity | 10.1016/j.jaci.2020.08.010 | 5 |
| American Academy of Allergy, Asthma & Immunology Needs Assessment Survey: Transitioning Primary Immunodeficiency Patients from Pediatric to Adult Care | 10.1016/j.jaci.2018.12.356 | 4 |
| Primary immunodeficiency: Potential benefits of active transition to improved multi-disciplinary care: Letter to the Editor | 10.1111/j.1399-3038.2008.00838.x | 5 |
| Burden of Poor Health Conditions and Quality of Life in 656 Children with Primary Immunodeficiency | 10.1016/j.jpeds.2017.10.029 | 4 |
| Into Action: Improving Access to Optimum Care for all Primary Immunodeficiency Patients | 10.1007/s10875-016-0277-2 | 5 |
| X-linked agammaglobulinemia (XLA): Phenotype, diagnosis, and therapeutic challenges around the world | 10.1016/j.waojou.2019.100018 | 4 |
| Psychosocial issues of the adolescent PI patient and the development of the PI Teen Outreach Program | 10.14785/lymphosign-2018-0002 | 4 |
| Growing older with a PID: transition of care and ageing. Recommendations of the International Patient Organisation for Primary Immunodeficiencies (IPOPI) | https://ipopi.org/wp-content/uploads/2017/06/IPOPI-recommendations-transition-of-care-and-ageing-FINAL_web.pdf | 5 |
| Improving the Transition from Pediatric to Adult Care for Adolescents and Young Adults with Autoinflammatory Diseases | 10.1007/978-3-319-96929-9_18 | 5 |
| Transition of pediatric patients with an Auto-inflammatory Disease: an alternative version of the Daedaulus and Icarus myth | 10.31138/mjr.29.3.156 | 5 |
| Transition from pediatric to adult care: Recommendations of the French network for autoimmune and autoinflammatory diseases (FAI2R) | 10.1016/j.revmed.2021.02.003 | 5 |
| Survey of attitudes of non-pediatric rheumatologists among councilors of the Japan College of Rheumatology regarding transitional care | 10.1080/14397595.2017.1285846 | 4 |
| Patient and parent perspectives on transition from paediatric to adult healthcare in rheumatic diseases: an interview study | 10.1136/bmjopen-2020-039670 | 4 |
| The readiness of pediatric rheumatology patients and their parents to transition to adult-oriented treatment | 10.1111/1756-185X.14050 | 4 |
| Recommendations for the management of autoinflammatory diseases | 10.1136/annrheumdis-2015-207546 | 5 |
| Current Research in Outcome Measures for Pediatric Rheumatic and Autoinflammatory Diseases | 10.1007/s11926-015-0558-4 | 5 |
