# Supplementary material for: Recommendations for Transitioning Young People with Primary Immunodeficiency Disorders and Autoinflammatory Diseases to Adult Care
Source: J Clin Immunol. 2024 Dec 17;45(1):57. doi: 10.1007/s10875-024-01838-y (PMC11652586; doi:10.1007/s10875-024-01838-y)
Supplement: Supplementary file 2 — Supplementary Table 2 (DOCX 16.2 KB) [file 10875_2024_1838_MOESM2_ESM.docx]

Supplementary Table 2. Proposed statements that did not achieve consensus.

| **Proposed statements that did not achieve consensus** | **Level of agreement** |
| --- | --- |
| The process of transition should ideally start when patients are between 12 and 14 years old.* | 57% |
| Young patients should have a period of simultaneous follow-up by the paediatric and adult clinics prior to transfer.* | 71% |
| Where possible, young patients should have a period of joint follow-up by the paediatric and adult clinics in the year following transfer when necessary. | 75% |
| Young patients without a diagnosis should not leave the paediatric service without a diagnostic and management re-evaluation.* | 70% |
| Young patients should have the opportunity for a (virtual) walk-through of the adult service to build familiarity with the site and its staff prior to transfer.* | 78% |
| Where possible, and in line with local legal frameworks, digital tools should be incorporated for smooth transition:   1. Develop smartphone or computer apps for patients to use as an ‘Electronic Health Record’ of disease activity, complications, hospitalisations, treatments, and adherence. 2. Use text messages or emails to communicate with young patients - as opposed to postal mail. | 72%  77% |
| The transition process should offer young patients the opportunity to interact with other patients of the same age(/peers with the same disease) by:   1. Holding special clinics for young people (Young adult clinics) 2. Occasionally organising social activities that introduce patients of the same age to each other | 70%  71% |
| Young adult patients, their family, and multidisciplinary clinicians should collaboratively develop a personalised transition plan that is informed by assessments of transition readiness (using transition tools such as the TRAQ, Good2Go etc. [53,54] | 70% |
| During the transition process, routine assessments should be performed using standardised measures (where available) of:   1. Transition readiness (using measures like TRAQ, Good2Go etc [53,54] 2. Service satisfaction 3. Educational/Vocational support needs (using measures like the Work and Social Adjustment Scale – Youth version and Work and Social Adjustment Scale – Parent version [55] | 65%  75%  71% |
| Patients should be advised to review private health care in countries where this is required to access adult care. | 78% |

* Statements that achieved consensus following revision in Round Three of the Delphi process
