# Supplementary material for: Recommendations for Transitioning Young People with Primary Immunodeficiency Disorders and Autoinflammatory Diseases to Adult Care
Source: J Clin Immunol. 2024 Dec 17;45(1):57. doi: 10.1007/s10875-024-01838-y (PMC11652586; doi:10.1007/s10875-024-01838-y)
Supplement: Supplementary file 3 — Supplementary Fig. 1 (DOCX 16 KB) [file 10875_2024_1838_MOESM3_ESM.docx]

Supplementary Figure 1. Number of patients transferred to adult services each year.
