# Supplementary material for: Recommendations for Transitioning Young People with Primary Immunodeficiency Disorders and Autoinflammatory Diseases to Adult Care
Source: J Clin Immunol. 2024 Dec 17;45(1):57. doi: 10.1007/s10875-024-01838-y (PMC11652586; doi:10.1007/s10875-024-01838-y)
Supplement: Supplementary file 4 — Supplementary Fig. 2 Heatmaps for all statements where agreement was >80% and <90%, sorted by responder category (left and column). The following categories are shown: (a) Disease category cared for: purple = autoinflammatory disease, dark blue = primary immunodeficiency, light blue = both (b) Age of patient seen: purple = adults, dark blue = children, light blue = both (c) Number of patients transferred to adult care per year: purple = <5, light grey = 5 to 10, light blue = >10; dark grey= unknown (d) Country representation: purple = poorly represented with 1-2 responders; better represented with >/= 3 responders. For (a)-(d): Top panels: rows represent individual responders; columns represent individual questions. Responses are coloured as follows: strongly agree = dark green; tend to agree = light green; neither agree nor disagree= yellow; tend to disagree= orange; strongly disagree=red; white = question not answered. Bottom panels: rows represent average response score for responders divided by specific categories represented in the top panels. Average response scored are coloured as follows: strongly agree = dark green; tend to agree = light green; neither agree nor disagree= yellow; tend to disagree= orange; strongly disagree=red. Strongly agree = average score for the category >/= 1.5; tend to agree = average score 1.49 to 0.5; neither agree nor disagree = average score 0.49 to -0.49; tend to disagree = average score -0.5 to -1.49; strongly disagree= average score </= -1.5. Statistical analysis for category comparison used the t-test; p<0.05 considered statistically significant. (DOCX 188 KB) [file 10875_2024_1838_MOESM4_ESM.docx]

1. Responses by disease category cared for

1. Responses by age of patient seen

1. Responses by number of patients transferred to adult care per year

1. Responses by country representation
